# RISK OF DIABETES AMONG ADULTS AGING WITH VISION IMPAIRMENT: THE ROLE OF THE NEIGHBORHOOD ENVIRONMENT

**DOI:** 10.1093/geroni/igac059.1013

**Published:** 2022-12-20

**Authors:** Philippa Clarke, Anam Khan, Kenzie Mintus, Joshua Ehrlich

**Affiliations:** University of Michigan, Ann Arbor, Michigan, United States; University of Michigan, Ann Arbor, Michigan, United States; Indiana University Purdue University Indianapolis, Indianapolis, Indiana, United States; University of Michigan, Ann Arbor, Michigan, United States

## Abstract

Experiencing vision loss as a result of the aging process may be different from aging with vision impairment (VI) acquired earlier in life. Adults aging with VI may be at risk for Type-2 Diabetes (T2DM) due to community barriers in accessing health care, healthy food, and recreational resources. We examined the relationship between neighborhood characteristics and incident diabetes in 22,719 adults aging with VI (without prevalent T2DM) in a private medical claims database (2008-2019). The primary outcome was time to incident T2DM diagnosis over 3+ years of enrollment. Cox models estimated hazard ratios (HRs) for incident diabetes (adjusted for age, sex, and comorbidities). Residence in neighborhoods with greater intersection density (HR=1.26) and traffic (HR=1.22) increased risk of T2DM, while broadband internet access (HR=0.67), optical stores (HR=0.62), supermarkets (HR=0.78), and gyms/fitness centers (HR=0.63) were associated with reduced risk. Results emphasize the importance of neighborhood context for aging well with VI.

